# Inhibition of BET Protein Function Suppressed the Overactivation of the Canonical NF-κB Signaling Pathway in 6-OHDA-Lesioned Rat Model of Levodopa-Induced Dyskinesia

**DOI:** 10.3389/fnins.2022.896322

**Published:** 2022-06-21

**Authors:** Ying Wan, Li Han, Lu Rong, Shuyuan Yang, Lu Song, Na Wu, Zhenguo Liu, Jing Gan

**Affiliations:** ^1^Department of Neurology, Xinhua Hospital Affiliated to Shanghai Jiao Tong University School of Medicine, Shanghai, China; ^2^Department of Gerontology, Xinhua Hospital Affiliated to Shanghai Jiao Tong University School of Medicine, Shanghai, China

**Keywords:** levodopa induced dyskinesia, inhibition of BET protein function, NF-κB signaling, neuroinflammation, intervention

## Abstract

**Background:**

Neuroinflammation is involved in the mechanisms of levodopa-induced dyskinesia (LID). The canonical NF-κB activation signaling pathway plays a critical role in the neuroinflammation development and BET protein-induced NF-κB-mediated neuroinflammation. The inhibition of the BET protein function has been reported to alleviate LID; however, its association with the canonical NF-κB signaling pathway in the 6-OHDA-lesioned striatum of the LID rat model remains unknown. Accordingly, we identified the status of the canonical NF-κB signaling pathway in the 6-OHDA-lesioned striatum of the LID rat model and whether the anti-dyskinetic effect of the BET inhibitor JQ1 was associated with its suppression on NF-κB-mediated neuroinflammation.

**Methods:**

6-OHDA PD rat models were treated with either L-dopa plus JQ1 or L-dopa alone. L-dopa treatment was given for 2 weeks, and the JQ1 treatment was given for 3 weeks and was initiated a week prior to L-dopa treatment. As a control, the sham rats were treated with JQ1 or Veh for 3 weeks. The ALO AIM assessment and cylinder test were performed during the treatment. Glial activation markers, pro-inflammatory substances, and critical proteins in the canonical NF-κB signaling pathway were tested in the lesioned striatum after the final treatment.

**Results:**

JQ1 effectively alleviated LID without influencing motor improvement. In the lesioned striatum, L-dopa triggered an overactivation of the canonical NF-κB signaling pathway, with an increase in the phospho-IKKα/β, phospho-IκBα, and NF-κB nuclear translocation and its phosphorylation at Ser 536 and Ser 276 sites (*p* < 0.01 vs. sham group). L-dopa induced an overexpression of the pro-inflammatory substances of tumor necrosis factor (TNF)-α, interleukin (IL)-1β, IL-6, and inducible nitric oxide synthase (iNOS), and the glial activation markers CD68 and GFAP. All the molecular changes were greatly inhibited by JQ1.

**Conclusion:**

L-dopa triggered an overactivation of the canonical NF-κB signaling pathway, leading to an enhanced neuroinflammation response in the 6-OHDA-lesioned striatum of LID rat models. The inhibition of the BET protein function significantly suppressed the activation of the canonical NF-κB signaling pathway in the striatum, alleviating the neuroinflammation response and the severity of LID.

## Introduction

Parkinson’s disease is a common global disabling neurodegenerative disease in the elderly population, characterized by cardinal motor symptoms of tremor, rigidity, bradykinesia, and postural instability. Its pathological hallmark is the loss of pigmented dopaminergic neurons in the substantia nigra (Fearnley and Lees, 1991). The decreased dopamine level in the striatum paralleled the severity of Parkinson’s motor symptoms. Oxidative stress is closely related to the pathology of PD (Trist et al., 2019), and in the DA-denervated striatum, microglia and astrocytes are pathologically activated, with an increased level of pro-inflammatory substances, leading to a chronic inflammatory status that results in neurodegeneration in PD (Ardah et al., 2020; Thadathil et al., 2021).

Levodopa (L-dopa) replacement therapy effectively ameliorates parkinsonian motor symptoms. However, with the increase in disease progression and L-dopa dosage, L-dopa-induced dyskinesia (LID) frequently occurs and impairs the clinical benefit of the medical intervention (Warren Olanow et al., 2013). Intermittent L-dopa administration mainly contributes to the occurrence of LID; however, it remains a major L-dopa delivery method. A recent study reported that intermittent L-dopa administration induced an enhanced neuroinflammation response in the striatum, compared to continuous L-dopa delivery (Mulas et al., 2016). Our recent study reported that the dyskinetic movement severity was significantly exacerbated when the striatal neuroinflammation response was enhanced by a systemic inflammatory stimulus of LPS prior to intermittent L-dopa administration (Yan et al., 2021). By contrast, the dyskinetic movements were greatly reduced in the PD model where the striatal neuroinflammation response was inhibited by a systemic administration of corticosterone prior to intermittent L-dopa stimulation (Barnum et al., 2008). Moreover, a recent study reported that aged PD rats presented with more serious dyskinetic movements and enhanced neuroinflammation response in their lesioned striatum than adult PD rats, despite a similar striatal dopamine loss level and the same L-dopa stimulus (Lanza et al., 2019). Increasing evidence supports a positive correlation between the neuroinflammation response in the striatum and the intensity of LID. Hence, neuroinflammation modulation might be a potential intervention for LID.

Nuclear factor (NF)-κB plays a critical role in neuroinflammation development. Physically, NF-κB is located in the cytoplasm. Once activated by a specific stimulus, it transfers to the nucleus and binds to the NF-κB response element, inducing pro-inflammatory gene expression (Perkins, 2007). There are several distinct NF-κB activation pathways, among which the canonical NF-κB activation signaling pathway responds to a variety of stimuli (Perkins, 2007). It is characterized by the activation of the IκB kinase complex (IKKα/β), IκBα phosphorylation, and subsequent degradation, as well as the nuclear translocation of NF-κB compounds (p50–p65). In addition, activated IKKα/β further promotes the phosphorylation of NF-κB (p65), which is dispensable for NF-κB transactivation (Fang et al., 2014; Lunin et al., 2019). Currently, there has been no report on the status of the canonical NF-κB signaling pathway in the 6-OHDA striatum of LID models.

Bromodomain and extra-terminal (BET) proteins belong to a family of proteins that contain two tandem bromodomains (BD1 and BD2) that bind to acetyl-lysine histone residues and non-histone proteins (Mandakini and Gregory, 2020). Through its unique structural features, BET proteins participate in the activation and elongation of transcription in mediating gene expression. Its family members include bromodomain-containing 2 (BRD2), BRD3, BRD4, and bromodomain testis associated protein (BRDT, only expressed in testis). Increasing studies have reported that BET proteins were involved in the canonical NF-κB activation pathway in autoinflammation disorders, injury, infection, and chronic morbid conditions, and inhibition of the BET protein function effectively suppresses the NF-κB-mediated pro-inflammatory expression, alleviating the neuroinflammation response in these pathological conditions (Jahagirdar et al., 2017; Wang et al., 2018; Sánchez-Ventura et al., 2019; Zhou et al., 2019). In the DA-denervated striatum of LID animal models, the inflammatory status is characterized by the overexpression of pro-inflammatory substances of tumor necrosis factor (TNF)-α, interleukin (IL)-1β, and inducible nitric oxide synthase (iNOS), which has been reported to be involved in the mechanisms of LID (Barnum et al., 2008; Bortolanza et al., 2015; Dos Santos Pereira et al., 2021). A recent study reported that a BET inhibitor JQ1 efficiently alleviated LID; however, its association with the impact of inhibition of the BET protein function on the NF-κB-mediated neuroinflammation in the striatum remains unknown.

Accordingly, we designed this study in which we aim to clarify (1) whether L-dopa induces the canonical NF-κB signaling pathway overactivation in the 6-OHDA-lesioned striatum of LID rat models and (2) whether the anti-dyskinetic effect of the BET inhibitor is associated with its suppression on the NF-κB-mediated neuroinflammation.

## Materials and Methods

### 6-Hydroxydopamine (6-OHDA)-Lesioned Parkinson’s Model

Adult male rats (Sprague–Dawley, 280–220 g) were used in our study. The procedures of the animal experiments were in accordance with the guidelines of the National Institutes of Health (publication No. 80–23) and were approved by the Institutional Review Board of Xinhua Hospital affiliated to the Shanghai Jiao Tong University Medical School. The model was designed as described previously (Wan et al., 2017). Intraperitoneal anesthetization with ketamine (100 mg kg^–1^, i.p.) was adopted. After anesthetization, the rats were placed on a stereotaxic frame (Narishige, Tokyo, Japan) to be injected with a 6-OHDA (Sigma Chemical Co., St. Louis, MO, United States) solution (6-OHDA in 0.9% saline and 0.02% ascorbic acid) at the right medial forebrain bundle (MFB) (6-OHDA concentration: 4 μg μl^–1^. 6-OHDA total dose: 32 μg rat^–1^). The two lesion coordinates were shown as follows: at AP –3.7 mm, ML +1.7 nm, DV –7.8 mm, and at AP –4.4 mm, ML +1.2 mm, DV –7.8 mm. The tooth bar was set to –2.4 mm. Each site was injected with 16 μg 6-OHDA per rat. A saline solution was injected into the same two sites of sham rats. At 3 weeks after injection, all injected rats were tested by the contralateral rotation assessment. 6-OHDA-lesioned rats with rotational behaviors (at least seven turns per minute) following apomorphine injection (i.p.) (Sigma Chemical Co., St. Louis, MO, United States) at a dose of 0.25 mg kg^–1^ were considered as successful rat models of PD, which were used in the following experiment.

### Drug Treatment and Behavioral Assessment

**FIGURE 1:**
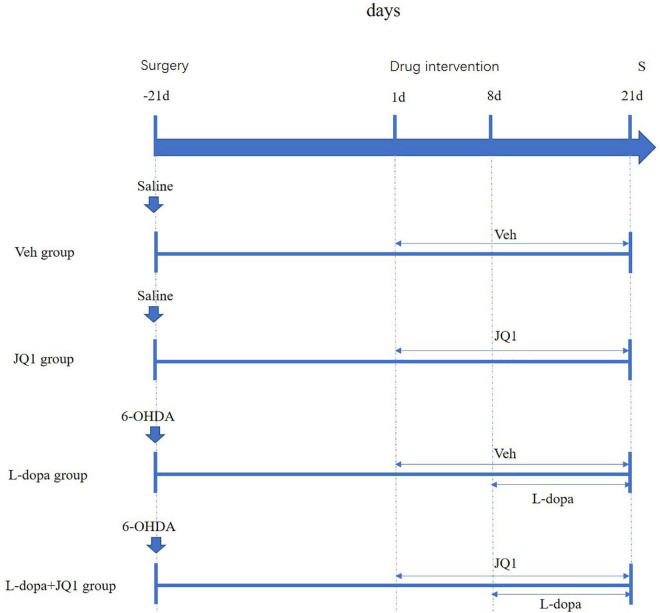
Schematic summarizing the experimental design. At 21 days after surgery, each group was administered with different intervention. Saline-lesioned sham rats were, respectively, treated with corresponding Veh (i.p.) (Veh group) or JQ1 (25 mg kg^–1^, i.p.) (JQ1 treatment group) once a day for 21 days; the 6-OHDA-lesioned PD rats were randomly divided into the L-dopa group and the L-dopa + JQ1 group. From day 1 to day 21, the two groups were correspondingly treated once daily (9 a.m.) with Veh or JQ1 (25 mg kg^–1^, i.p.) for 21 days; from day 8 to day 21, both the two groups were immediately treated with L-dopa (L-dopa, 12 mg kg^–1^, bensarazide, 6 mg kg^–1^) (i.p.) immediately after Veh or JQ1 administration. About 1 h after drug administration on day 21, all rats were anesthetized for scarification for tissue collection. Veh: an equal amount of DMSO diluted in carrier solution; S: scarification.

We prepared the JQ1 (a small BET inhibitor) (MedChem Express, New Jerk, United States) solution as described in a previous report (Song et al., 2019). A pure JQ1 powder was dissolved in the following order: 2% DMSO (Sigma, Chemical Co., St. Louis, MO, United States), 30% PEG300 (Sigma, Chemical Co., St. Louis, MO, United States), 5% Tween 80 (Sigma, Chemical Co., St. Louis, MO, United States), and ddH_2_0. The solution was freshly prepared before use. Drug administration was carried out as described in a previous report (Figge and Standaert, 2017). The successful PD rat models were randomly divided into three groups: (1) the L-dopa + JQ1 treatment group (*n* = 18): the PD rats were administered with both JQ1 solution and L-dopa. JQ1 administration lasted for 3 weeks [for the first week, the PD rats were only treated with the JQ1 (25 mg kg^–1^, i.p.) solution once a day (9 a.m.)], and for the remaining 2 weeks, the PD rats were treated with both JQ1 solution (25 mg kg^–1^, i.p., once a day, 9 a.m., immediately prior to the L-dopa administration) and L-dopa (L-dopa, 12 mg kg^–1^, i.p. and bensarazide, 6 mg kg^–1^, i.p. once a day, 9 a.m.); (2) L-dopa treatment group (*n* = 18): the PD rats were administered once daily (9 a.m.) with L-dopa (L-dopa, 12 mg kg^–1^, i.p. and bensarazide, 6 mg kg^–1^, i.p.) for 2 weeks. In addition, the rats of this group were given the vehicle that had an equal amount of DMSO diluted in carrier solution (i.p., once a day, 9 a.m.) for 3 weeks, which started 1 week prior to the initiation of L-dopa administration; and (3) PD group (*n* = 6): the PD rats were administered once daily (9 a.m.) with the vehicle for 3 weeks. This group was only used for the cylinder test. The sham rats were randomly divided into two groups: (4) the JQ1 treatment group (*n* = 18): sham rats were administered with the JQ1 (JQ1, 25 mg kg^–1^, i.p.) solution once a day (9 a.m.) for 3 weeks (5); the Veh treatment group (*n* = 18): the sham rats were administered with the vehicle (i.p., once a day, 9 a.m.) for 3 weeks. The experimental design is illustrated in Figure 1.

### Abnormal Involuntary Movement Ratings

All PD rats were tested by the Abnormal Involuntary Movement (AIM) assessments. AIM assessments were performed once a day on days 9, 13, 17, and 20. The detailed AIM rating system is described in the previous study (Wan et al., 2017). Generally, the assessment of AIMs was made up of three parts: the axial, the limb, and the orolingual movements (ALO AIMs) (Lindenbach et al., 2011). For each part of the AIMs, the severity was scored from 0 to 4 (Oh et al., 1999; Rylander et al., 2009). The theoretical maximum score of each AIM subtype was 24 points (Cenci et al., 1998). The theoretical maximum score of the total ALO AIM scores was 72 points. All the assessments were conducted by an investigator who was blind to the experiment.

### Cylinder Test

We used the cylinder test to assess the functional improvement of the pharmacological intervention on the rats’ impaired forelimb contralateral to the 6-OHDA-lesioned striatum (Schallert et al., 2000). The test was performed in the PD rats once a day on days 10, 14, and 18. In the cylinder test, the rats were individually placed in a transparent cylinder (20 cm diameter and 30 cm height) in a dimly lit room for 5 min. Such actions were recorded when the rat independently used its left or right forelimb to touch the wall during a full rear to start a weight-shifting movement or to regain center of gravity while moving laterally in a vertical posture. Functional improvement of the PD rat’s impaired forelimb was assessed by the percentage of use of the impaired forelimb relative to the total number of forelimb touching movements.

### RNA Isolation and Real-Time PCR

After anesthetization, the rats were killed by decapitation to prepare the total RNA extraction of lesioned striatum 1 h following the final treatment. The total RNA was acquired by using TRIzol reagent (Invitrogen, CA, United States). A measure o 1 μg of total RNA was reverse-transcribed to cDNA using 5 × PrimeScript RT Master Mix (Takara, Japan) in accordance with the manufacturer’s instruction. For PCR amplification, 10 mL reaction volume was used, including 5 mL of 2 × SYBR Premix Ex Taq mixture (Takara, Japan), 0.1 mmol/L of each primer, and 1 mL of twofold diluted cDNA and sterile distilled water. The reaction and detection were conducted in a light cycler (Roche, Mannheim, Germany). The 2^–ΔΔ^ ^Ct^ method was used to calculate the relative mRNA levels of each target gene. The primers usen for TNF-α, IL-1β, IL-6, and iNOS were listed as follows: *tnf-*α (F) 5′-GACACCATGAGCACGGAAAG-3′, (R) 5′-TCCTCTGCCAGTTCCACATC-3′, *Il-1*β (F) 5′-CTGCCA AGTCAGGTCTCTCA-3′, (R) 5′-CCACTGCCTTCCCTACTT CA, *Il-6* (F) 5′-CCACTGCCTTCCCTACTTCA-3′, (R) 5′-TGGT CTGTTGTGGGTGGTAT-3′, *inos* (F) 5′-ACCAGGAGGCGCCA TCCCGCTGC-3′, and (R) 5′-CTTGATCAAACACTCATTTT AT-3′. The cycle threshold (Ct) values were collected and normalized to the level of the housekeeping gene gapdh. The 2^–ΔΔ^ ^Ct^ method was used to calculate the relative mRNA levels of each target gene.

### ELISA

After anesthetization, the rats were killed by decapitation to acquire the lesioned striatum 1 h after the final drug administration. Each 100 μg striatal tissue was dissociated in 100 μl protein lysate (Beyotime, Shanghai, China) with the addition of protease inhibitor (Beyotime, Shanghai, China) at a ratio of 1:100. After tissue grinding, the mixed liquid was centrifugated at 12,000 rpm, 4°C for 10 min. The supernatant was collected for assay of TNF-α, IL-1β, IL-6, and iNOS by using ELISA kits (IL-1β and IL-6, Abcam, Shanghai, China) (TNF-α and iNOS, Elabscience, Wuhan, China) following the manufacturer’s instructions, respectively. Optical density was obtained at 450 nm using a microplate reader within 15 min of stop solution addition.

### Striatal Protein Extraction

After anesthetization, the rats were killed by decapitation 1 h after last injection on the final day, and the lesioned striatum was immediately dissected. The total protein was acquired as follows: each 100 μg striatal tissue was dissociated in 100 μl protein lysate (Beyotime, Shanghai, China) with the addition of protease inhibitor (Beyotime, Shanghai, China) at a ratio of 1:100. After tissue grinding, the mixed liquid was centrifugated at 12,000 rpm, 4°C for 10 min. The supernatant was collected and tested for the concentration of protein by using a BCA kit (Pierce, Rockford, United States). The cytoplastic protein was acquired as follows: the striatal tissue was cut into small fragments, and then 1 ml of protein extraction reagent A (Beyotime, Shanghai, China) which contains PMSF was added to the tissue fragments. The mixture was placed in ice for 10–15 min and then was centrifuged at 700 *g* at 4°C for 10 min. The supernatant was then carefully transferred to a new tube, centrifuged at 14,000 *g* at 4°C for 30 min to precipitate the cell membrane fragments. The supernatant containing cytoplasmic protein was collected, and then protein concentration was determined by using the BCA kit (Pierce, Rockford, United States). In terms of acquiring nuclear protein, the striatal tissue was cut into small fragments and was added to protein extraction reagents A and B at a ratio of 20:1 (Beyotime, Shanghai, China). Then the mixture was diluted in PMSF (1 mM) at the ratio of 60 mg tissue per 200 μl tissue homogenate and was homogenized at a pre-cooled homogenizer at 60 Hz for 180 s; then it was transferred to a new tube for centrifugation at 12,000*g*, 4°C for 3 min. The supernatant was removed; 50 μl of nuclear protein extraction reagent that contained PMSF to the precipitate was added, vortexed for 15–30 s, and then centrifuged at 12,000*g*, 4°C for 10 min. The supernatant of nuclear protein was immediately sucked into a precooled plastic tube. All the protein samples were sub-packaged and frozen at –80°C for future Western blot.

### Western Blot Analysis

Samples containing equivalent amounts of protein (30 μg) were electrophoresed on 10% sodium dodecyl sulfate–polyacrylamide gel. Proteins were electro-transferred to the polyvinylidene fluoride (PVDF) membrane for immunoblotting in Tris–glycine transfer buffer. Then, the membrane was blocked in a blocking buffer with 5% non-fat dry milk in TBS-Tween 20 for an hour at room temperature. The membrane was incubated at 4°C overnight with an antibody recognizing IKKα/β (1:1000) (Proteintech Group, Rosemont, United States); phospho-IKKα/β (1:500) (Signalway Antibody LLC, College Park, MD, United States); IκBα (1:1000) (Santa Cruz Technology, CA, United States); phospho-IκBα (1:1,000) (Cell Signaling, MA, United States); phospho-NF-κB (p65) (Ser 536) (1:1000) (Absin Bioscience, Shanghai, China); phospho-NF-κB (p65) (Ser276) (1: 1000) (Absin Bioscience, Shanghai, China); cytoplasmic NF-κB (1:1000) (Cell Signaling, MA, United States); nuclear NF-κB (1: 1000) (Cell Signaling, MA, United States); extracellular signal regulated kinase 1/2 (ERK1/2) (1:2,000) (Cell Signaling, MA, United States); phospho-ERK1/2 (1:1,000) (Cell Signaling, MA, United States); tyrosine hydroxylase (TH) (1:5000) (PTG, Chicago, United States); CD68 (1:2000) (Abcam, Boston, United States); glial fibrillary acidic protein (GFAP) (1:5000) (PTG, Chicago, United States); and tubulin (1:8000) (PTG, Chicago, United States). Subsequently, the membrane was washed extensively with TBS-T and incubated with horseradish peroxidase-conjugated (HRP-conjugated) anti-rabbit or anti-mouse IgG (1:2,000) (Cell Signaling, MA, United States) for an hour at room temperature. Visualization of immunoreactive proteins was achieved by using the enhanced chemiluminescence detection system (Millipore). Bands of interest were analyzed quantitatively using Image Lab software (Bio-Rad).

### Statistical Analysis

Data were displayed as mean ± standard deviation (SD). The independent *T*-test was used for comparison between two groups. One-way analysis of variance was used for comparison of behavior and biochemical data among four groups. The *post hoc* test was conducted by using the LSD test. Statistical significance was set at *p* < 0.05.

## Results

### Inhibition of BET Protein Function Reduced ALO Abnormal Involuntary Movement Scores of 6-OHDA-Lesioned Rats Treated With L-Dopa

**FIGURE 2:**
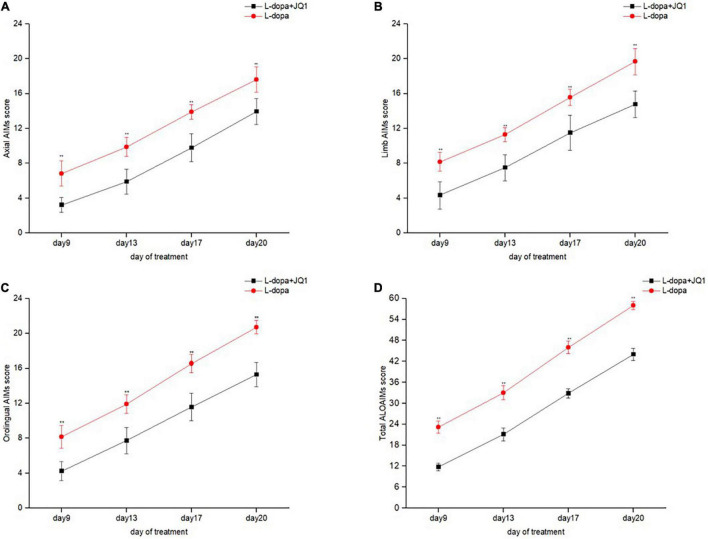
Dyskinetic movement assessment of the 6-OHDA-lesioned PD rats during the treatment with L-dopa (L-dopa, 12 mg kg^–1^, bensarazide, 6 mg kg^–1^, i.p., once a day) (L-dopa group, *n* = 18) and L-dopa (L-dopa, 12 mg kg^–1^, bensarazide, 6 mg kg^–1^, i.p., once a day) plus JQ1 (25 mg kg^–1^, i.p., once a day) (L-dopa + JQ1 treatment group, *n* = 18). Measures of axial **(A)**, limb **(B)**, orolingual **(C)**, and the total ALO AIMs **(D)** in the rats treated with L-dopa and L-dopa + JQ1 on each assessment during the 21 days of treatment. During the period, the 6-OHDA-lesioned rats in the PD + L-dopa group showed a robust increase in the scores of the total ALO AIM assessment and each of the subitems, with apparent dyskinetic movements. By contrast, this phenomenon was apparently inhibited in 6-OHDA-lesioned PD rats treated with L-dopa plus JQ1 (L-dopa + JQ1 treatment group). The scores of total ALO AIM assessment and its subitems in the L-dopa treatment group were all significantly higher than in the L-dopa + JQ1 treatment group at each assessment (*p* < 0.001). Data are presented as mean ± SD; **p < 0.001 vs. PD + L-dopa group.

We compared the ALO AIM scores between two treatment groups to observe JQ1 effect on the severity of LID. As shown in Figure 2, compared with the L-dopa treatment group, the total and subgroup scores of ALO AIM assessment in the L-dopa + JQ1 treatment group were significantly lower at each test, which supported an anti-dyskinetic role of JQ1 (*p* < 0.001 vs. L-dopa treatment group).

### Inhibition of BET Protein Function Had No Effect on the Motor Improvement in the 6-OHDA-Lesioned PD Rats Treated With L-Dopa

**FIGURE 3:**
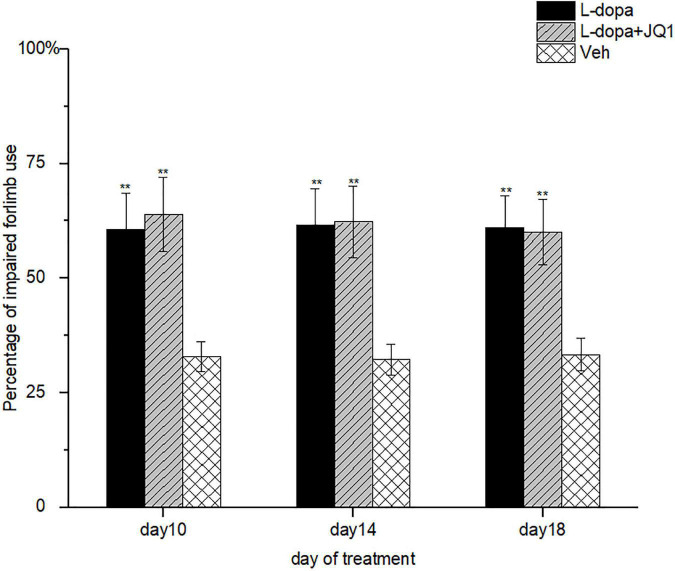
Motor improvement of 6-OHDA-lesioned rat models of PD treated with L-dopa (L-dopa, 12 mg kg^–1^, bensarazide, 6 mg kg^–1^, i.p., once a day) (L-dopa treatment group, *n* = 18), L-dopa (L-dopa, 12 mg kg^–1^, bensarazide, 6 mg kg^–1^, i.p., once a day) plus JQ1 (25 mg kg^–1^, i.p., once a day) (L-dopa + JQ1 treatment group, *n* = 18), or Veh (*n* = 6) was assessed by cylinder tests on days 10, 14, and 18 of the treatment. At each assessment, both the L-dopa group and the L-dopa + JQ1 group showed a better performance as the treated 6-OHA-lesioned PD rats preferred to touch the inner wall of the cylinder with the impaired forelimbs more frequently (*p* < 0.001 vs. Veh group). Meanwhile, no difference was found in the rats’ preference in touching the inner wall with the impaired forelimbs in cylinder tests between the L-dopa and L-dopa + JQ1 treatment groups (*p* > 0.05). Data are presented as mean ± SD; **p < 0.001 vs. Veh treatment.

It remained unknown whether the inhibition of the BET protein function interferes with motor improvement induced by L-dopa. Therefore, we assessed the motor function of impaired forelimb contralateral to the lesioned striatum of treated PD rats using the cylinder test. As shown in Figure 3, at each assessment, the PD rats in both the L-dopa and L-dopa + JQ1 treatment groups displayed an increased times of touching to the wall with its impaired forelimbs (*p* < 0.001 vs. the PD group). No difference was found in the motor improvement between the two treatment groups (*p* > 0.05).

### Inhibition of BET Protein Function Barely Affected Striatal Tyrosine Hydroxylase Expression

**FIGURE 4:**
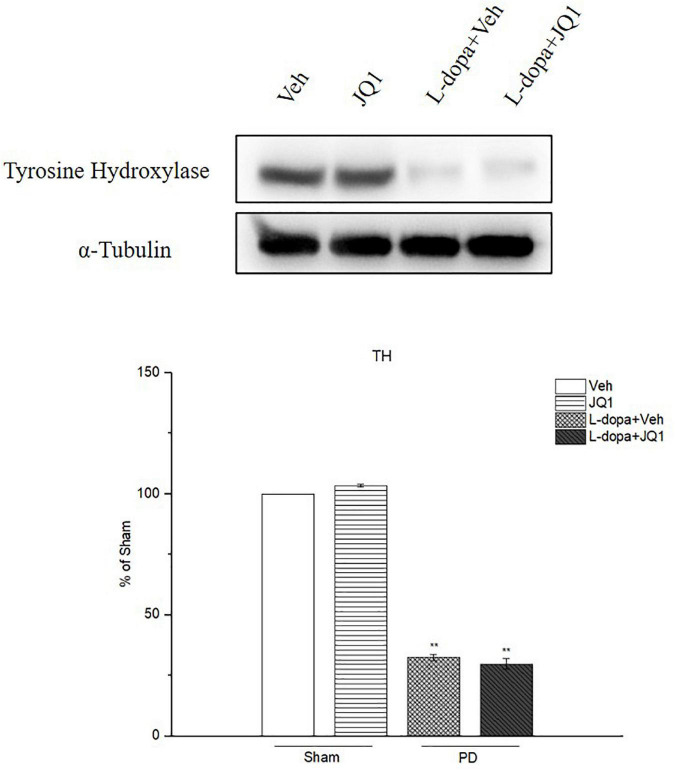
Changes in the TH protein level extracted from the saline- or 6-OHDA-lesioned striatum after 21 days of administration. Both L-dopa treatment group and L-dopa + JQ1 treatment groups showed a significant reduction in the TH protein level in the rats’ ipsilateral striatum after 21 days of treatment (*p* < 0.001 vs. Veh group). No difference was found in the TH protein level between L-dopa and L-dopa + JQ1 treatment groups (*p* > 0.05). TH levels expression relative to α-tubulin levels. The data are expressed in terms of mean ± SD and as a percentage of the Veh group. **p < 0.001 vs. Veh group (*n* = 3 per group).

By using Western blot, we confirmed a decreased TH protein level in the 6-OHDA-lesioned PD rat model treated with L-dopa and L-dopa + JQ1 (Figure 4). It indicated that 6-OHDA significantly caused the striatal dopaminergic terminal fiber loss and motor impairment contralateral to the lesions. L-dopa effectively improved the motor function. Moreover, the TH protein level in the JQ1 treatment group was same as that in the Veh group (*p* > 0.05). Accordingly, JQ1 had no protective or toxic effect on the striatal dopaminergic terminal fibers.

### Inhibition of BET Protein Function Inhibited ERK1/2 Hyperphosphorylation in the 6-OHDA-Lesioned Striatum of PD Rats Treated With L-Dopa

**FIGURE 5:**
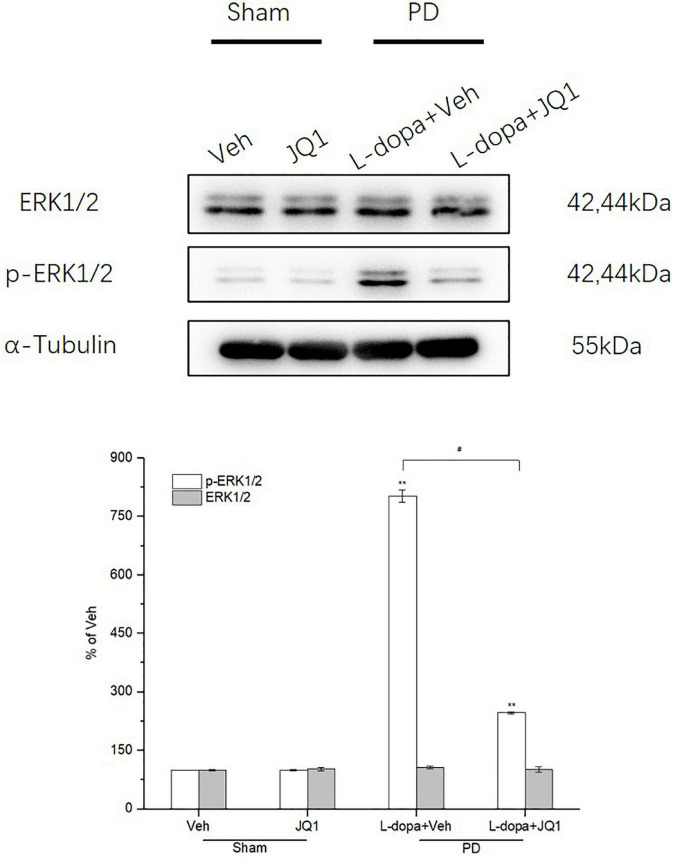
Changes in the expression of ERK1/2 and its phosphorylation extracted from the saline- or 6-OHDA-lesioned striatum after 21 days of administration. Both the L-dopa and L-dopa + JQ1 treatment groups showed an aberrant increase in the protein level of phoshpo-ERK1/2 in the 6-OHDA-lesioned striatum of rats after 21 days of treatment (*p* < 0.001 vs. Veh group). Moreover, the increase in phospho-ERK1/2 in the L-dopa treatment group was greater than that in the L-dopa + JQ1 treatment group (*p* < 0.01). In addition, JQ1 barely affected the expression of phospho-ERK1/2 in the saline-lesioned striatum in the JQ1 treatment group (*p* > 0.05 vs. Veh group). The ERK1/2 protein level remained unchanged among the four treatment groups. All the aforementioned protein levels expressed relative to α-tubulin levels. The data are expressed in terms of mean ± SD and as a percentage of the Veh group. **p < 0.001 vs. Veh group, ^#^*p* < 0.01 vs. L-dopa treatment group (*n* = 3 per group).

ERK1/2 phosphorylation is a critical molecular event in the mechanisms of LID. We observed whether inhibition of the BET protein function affected ERK1/2 phosphorylation through Western blot. As shown in Figure 5, in the L-dopa treatment group, we detected a robust increased phospho-ERK1/2 level in the 6-OHDA-lesioned striatum of rats with serious dyskinetic movements; however, in the L-dopa + JQ1 treatment group, phospho-ERK1/2 increased mildly in the lesioned striatum of rats with mild dyskinesia (*p* < 0.001 vs. the Veh group, *p* < 0.01 vs. L-dopa treatment group). Phospho-ERK1/2 expression remained unchanged in the JQ1 treatment group (*p* > 0.05 vs. the Veh group). The total ERK1/2 expression remained unchanged among the four treatment groups (*p* > 0.05 vs. Veh group).

### Inhibition of BET Protein Function Suppressed the Neuroinflammation Response in the 6-OHDA-Lesioned Striatum of PD Rats Treated With L-Dopa

**FIGURE 6:**
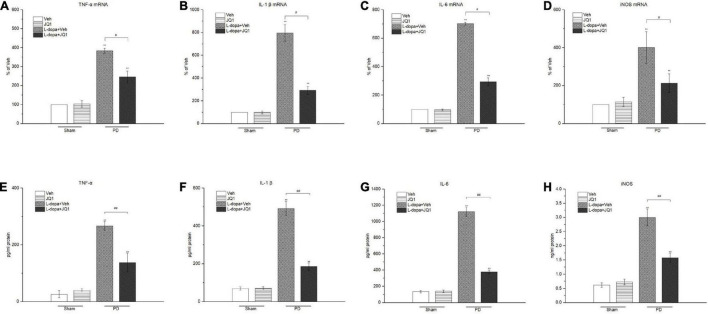
Changes in the mRNA **(A–D)** and protein **(E–H)** levels of pro-inflammatory mediators extracted from the saline- or 6-OHDA-lesioned striatum after 21 days of administration. Both L-dopa and the L-dopa + JQ1 treatment groups showed an aberrant increase in both the mRNA and protein levels of the TNF-α, IL-1 β, IL-6, and iNOS in the 6-OHDA-lesioned striatum of rats after the 21 days of treatment (*p* < 0.001 vs. Veh group). Moreover, the expression level in the L-dopa treatment group was much higher than that in the L-dopa + JQ1 treatment group (*p* < 0.01). JQ1 barely affected the expression level of the four pro-inflammatory substances in the saline-lesioned striatum of the JQ1 treatment group (*p* > 0.05 vs. Veh group). The data are expressed in terms of mean ± SD and as a percentage of the Veh group, **p < 0.001 vs. Veh group; ^#^*p* < 0.01 vs. L-dopa treatment group, ^##^*p* < 0.001 vs. L-dopa treatment group (*n* = 4 per group for RT-PCR; *n* = 5 per group for ELISA).

**FIGURE 7:**
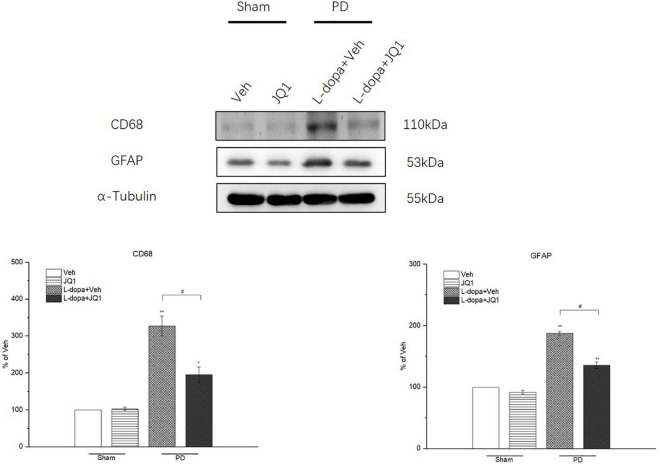
Changes in the CD68 and GFAP expressions in the saline- or 6-OHDA-lesioned striatum after 21 days of administration. Both L-dopa and L-dopa + JQ1 treatment groups showed an aberrant increase in the protein level of CD68 and GFAP in the 6-OHDA-lesioned striatum of rats after 21 days of treatment (*p* < 0.01 vs. Veh group). The protein level of CD68 and GFAP in the L-dopa treatment group was much higher than that in the L-dopa + JQ1 treatment group (*p* < 0.01). JQ1 barely affected the CD68 and GFAP protein expressions in the saline-lesioned striatum of the JQ1 treatment group (*p* > 0.05 vs. Veh group). The protein levels expressed relative to α-tubulin levels. The data are expressed in terms of mean ± SD and as a percentage of the Veh group. **p < 0.001 vs. Veh group, *p < 0.01 vs. Veh group, ^#^*p* < 0.01 vs. L-dopa treatment group (*n* = 3 per group).

For identification of the effect of inhibition of the BET protein function on the striatal neuroinflammation response, we used the RT-PCR method to test the mRNA levels of the pro-inflammatory substances of TNF-α, IL-1β, IL-6, and iNOS in different treatment groups. As shown in Figures 6A–D, an increase was observed in the four mRNA levels in the 6-OHDA-lesioned striatum from both the L-dopa and L-dopa + JQ1 treatment groups (*p* < 0.001 vs. the Veh group); however, JQ1 significantly suppressed such an increase in the L-dopa + JQ1 treatment group (*p* < 0.01 vs. L-dopa group). Furthermore, by using ELISA, we detected an aberrant increase in the four proteins in both the L-dopa and L-dopa + JQ1 treatment groups (*p* < 0.001 vs. Veh). JQ1 significantly suppressed such an increase in the L-dopa + JQ1 treatment group (*p* < 0.001 vs. L-dopa group), which is shown in Figures 6E–H. The mRNA and protein levels of the four pro-inflammatory substances remained unchanged in the JQ1 treatment group (*p* > 0.05 vs. the Veh group). We further compared the glial activation in the 6-OHDA-lesioned striatum among different treatment groups by Western blot. As shown in Figure 7, the L-dopa treatment group expressed a robust increase in the protein level of CD68 (microglial activation) and GFAP (astrocyte activation) (*p* < 0.001 vs. the Veh group). By contrast, in the L-dopa + JQ1 group, we detected a mild increase in the two protein levels in the 6-OHDA-lesioned striatum of rats with mild dyskinesia movements (*p* < 0.001 vs. the Veh group, *p* < 0.01 vs. L-dopa group). The protein level of CD68 and GFAP remained unchanged in the JQ1 treatment group (*p* > 0.05 vs. the Veh group).

### Inhibition of BET Protein Function Suppressed the Phosphorylation of IKKα/β and IκBα and Degradation of IκBα in the 6-OHDA-Lesioned Striatum of PD Rats Treated With L-Dopa

**FIGURE 8:**
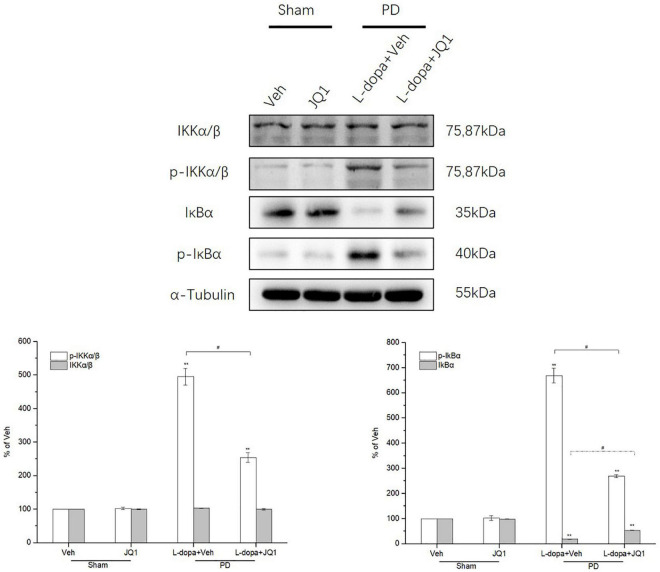
Changes in the expression of IKKα/β, phospho-IKKα/β, IκBα, and phospho-IκBα in the saline- or 6-OHDA-lesioned striatum after 21 days of administration. Intermittent L-dopa stimulation induced a significant increase in the protein level of phospho-IKKα/β and phospho-IκBα in the 6-OHDA-lesioned striatum in both L-dopa and L-dopa + JQ1 treatment groups (*p* < 0.001 vs. Veh group). The protein level of phospho-IKKα/β and phospho-IκBα in the L-dopa treatment group was much higher than that in the L-dopa + JQ1 treatment group (*p* < 0.01). Notably, intermittent L-dopa stimulation induced an apparent reduction in the protein level of IκBα in both the L-dopa and L-dopa + JQ1 treatment groups (*p* < 0.001 vs. Veh group). The protein level of IκBα in the L-dopa treatment group was statistically lower than that in the L-dopa + JQ1 treatment group (*p* < 0.01). JQ1 barely affected the expression of phospho-IKKα/β, phospho-IκBα, and IκBα in the saline-lesioned striatum of JQ1 treatment group. The IKKα/β protein level remained unchanged among the four treatment groups. The protein levels expressed relative to α-tubulin levels. The data are expressed in terms of mean ± SD and as a percentage of the Veh group, **p < 0.001 vs. Veh group, ^#^*p* < 0.01 vs. L-dopa treatment group (*n* = 3 per group).

We observed whether L-dopa triggered the overactivation of the canonical NF-κB pathway in the lesioned striatum by testing the total and phosphorylation protein levels of IKKα/β and IκBα by Western blot. As shown in Figure 8, L-dopa triggered an overactivation of IKKα/β and IκBα in the lesioned striatum with an increased phospho-IKKα/β and phospho-IκBα protein levels (*p* < 0.001 vs. the Veh group). The total IκBα protein level greatly decreased in the L-dopa treatment group (*p* < 0.001 vs. the Veh group). In the L-dopa + JQ1 treatment group, JQ1 significantly inhibited the overactivation of the canonical NF-κB pathway, with a less increased phospho-IKKα/β and phospho-IκBα protein levels and a mild reduction in the total IκBα protein level (*p* < 0.001 vs. the Veh group, *p* < 0.01 v L-dopa treatment group). The IKKα/β protein level remained unchanged among the four groups, and JQ1 barely affected the IκBα protein level in the JQ1 treatment group (*p* > 0.05 vs. the Veh group).

### Inhibition of BET Protein Function Blocked the NF-κB Nuclear Translocation

**FIGURE 9:**
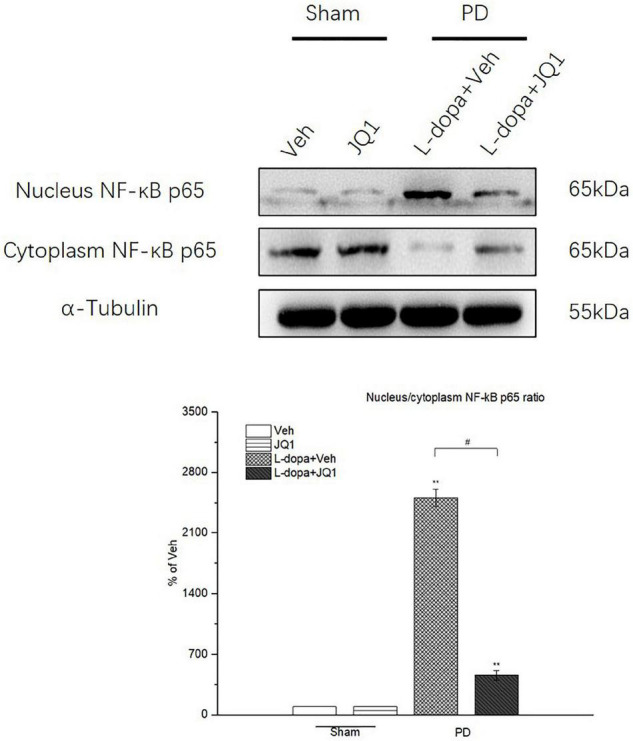
Changes in the ratio of nuclear NF-κB p65 to cytoplasmic NF-κB p65 in the saline- or 6-OHDA-lesioned striatum after 21 days of administration. Intermittent L-dopa stimulation induced a significant increase in the ratio of nuclear NF-κB p65 to cytoplasmic NF-κB p65 in the 6-OHDA-lesioned striatum from both L-dopa and L-dopa + JQ1 treatment groups (*p* < 0.001 vs. Veh group). Moreover, the ratio of nuclear NF-κB p65 to cytoplasmic NF-κB p65 in L-dopa treatment group was statistically higher than that in the L-dopa + JQ1 treatment group (*p* < 0.001). JQ1 barely affected the ratio in the saline-lesioned striatum in the JQ1 treatment group. The protein levels expressed relative to α-tubulin levels. The data are expressed in terms of mean ± SD and as a percentage of the Veh group. **p < 0.001 vs. Veh group; ^#^*p* < 0.01 vs. L-dopa treatment group (*n* = 6 per group, the repetition no. = 3).

In the canonical NF-κB activation pathway, NF-κB-mediated neuroinflammation was characterized by the nuclear translocation of NF-κB p65. Therefore, we tested the protein level of cytoplasmic and nuclear NF-κB p65 by Western blot and compared the ratio of nuclear NF-κB p65 to cytoplasmic NF-κB p65 among different treatment groups. As shown in Figure 9, in the L-dopa treatment group, a high value was observed in the ratio of nuclear NF-κB p65 to cytoplasmic NF-κB p65 (*p* < 0.001 vs. the Veh group). By contrast, JQ1 significantly inhibited the ratio increase in the L-dopa + JQ1 treatment group (*p* < 0.001 vs. the Veh group; *p* < 0.01 vs. the L-dopa treatment group). The ratio remained unchanged in the JQ1 treatment group (*p* > 0.05 vs. the Veh group).

### Inhibition of BET Protein Function Suppressed the Phosphorylation of NF-κB p65 at Ser 536 and Ser 276 in the 6-OHDA-Lesioned Striatum of PD Rats Treated With L-Dopa

**FIGURE 10:**
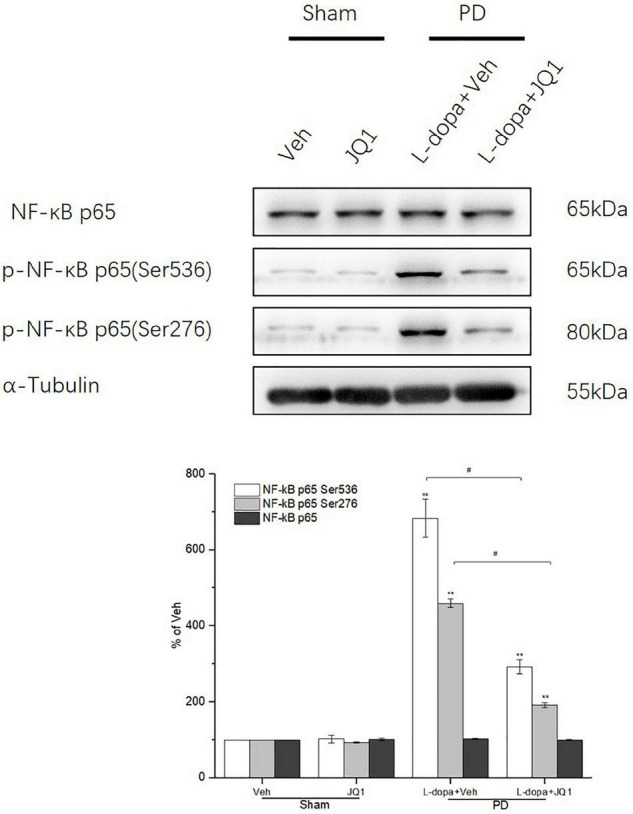
Changes in the expression of NF-κB p65 and its phosphorylation (Ser 536, Ser 276) extracted from the saline- or 6-OHDA-lesioned striatum after 21 days of administration. Both the L-dopa group and the L-dopa + JQ1 treatment groups showed an aberrant increase in the protein level of phospho-NF-κB p65 (Ser 536 and Ser276) in the 6-OHDA-lesioned striatum of rats after 21 days of treatment (*p* < 0.001 vs. Veh group). Moreover, the increase in phospho-NF-κB p65 at the two sites in the L-dopa treatment group was greater than in the L-dopa + JQ1 treatment group (*p* < 0.01). In addition, JQ1 barely affected the expression of phospho-NF-κB p65 (Ser 536 and Ser276) in the saline-lesioned striatum in JQ1 treatment group (*p* > 0.05 vs. Veh group). The NF-κB p65 protein level remained unchanged among the four treatment groups. All the aforementioned protein levels expressed relative to α-tubulin levels. The data are expressed in terms of mean ± SD and as a percentage of the Veh group, **p < 0.001 vs. Veh group, ^#^*p* < 0.01 vs. L-dopa treatment group (*n* = 3 per group).

In the canonical NF-κB activation pathway, phosphorylation of NF-κB p65 at Ser 536 and Ser276 sites is crucial for NF-κB transactivation. Therefore, we tested Ser 536 and Ser276 phosphorylation levels in the four groups to observe its change under different interventions. As shown in Figure 10, in the L-dopa treatment group, we observed an aberrant increase in Ser 536 and Ser276 phosphorylation in the 6-OHDA-lesioned striatum of rats with serious dyskinesia (*p* < 0.001 vs. the Veh group). In the L-dopa + JQ1 treatment group, there was a mild increase in the rats with mild dyskinesia (*p* < 0.001 vs. Veh; *p* < 0.01 vs. L-dopa treatment group). The phosphorylation level at the two sites remained unchanged in the JQ1 treatment group (*p* > 0.05 vs. the Veh group). The total p65 protein level remained unchanged among the four treatment groups (*p* > 0.05 vs. the Veh group).

## Discussion

Our results indicated that intermittent L-dopa stimulation induced overactivation of the canonical NF-κB signaling pathway in the 6-OHDA-lesioned striatum of LID rat models. The inhibition of the BET protein function significantly suppressed the overactivation of the canonical NF-κB signaling pathway, reducing neuroinflammation response triggered by intermittent L-dopa stimulation and alleviating the intensity of LID. Accordingly, we proposed that the inhibition of the BET protein function might play an anti-dyskinetic role, which was associated with its anti-inflammation function through blocking the overactivation of the canonical NF-κB signaling pathway.

NF-κB activation is a core event in inflammation development, which is mainly mediated through the canonical and non-canonical NF-κB activation signaling pathways (Perkins, 2007). The canonical NF-κB activation signaling pathway widely and rapidly responds to various inflammatory stimuli, including pro-inflammatory cytokines of TNF-α, IL-1β, etc., (Liu et al., 2017) which activates IKKα/β. Activated IKKα/β further phosphorylates IκBα and thereby triggers ubiquitin-dependent IκBα degradation in the proteasome, resulting in rapid and transient nuclear translocation of NF-κB (p50–p65). Within the nucleus, NF-κB binds to the DNA *cis-*regulatory elements at enhancers and promoters, prompting pro-inflammatory gene expression. During this process, nuclear NF-κB p65 was acetylated by p300 at the lysin 310 site and then interacted with BET proteins, promoting the following recruitment of the positive transcription elongation complex (P-TEFb) and chromatin remodeling factors for the targeted gene transcription (Huang et al., 2009; Hajmirza et al., 2018). We speculated that the intermittent L-dopa stimulation aggravated the oxidative stress burden in the 6-OHDA-lesioned striatum, leading to the overexpression of TNF-α and IL-1β, which further induced the overactivation of the canonical NF-κB signaling pathway in a vicious cycle. The non-canonical NF-κB activation pathway, which is characterized by the activation of kinase NF-κB-inducing kinase (NIK) and the nuclear translocation of NF-κB p52, selectively responses to a subset of TNF receptor (TNFR) superfamily members and mainly participates in the regulation of lymphoid organ development, B-cell survival, and maturation of the differentiation of osteoclasts (Sun, 2017). There is a slim possibility that the non-canonical NF-κB activation signaling pathway is involved in the neuroinflammation development in the neurodegenerative disorders; however, one study reported that both canonical and non-canonical NF-κB activation signaling pathways were induced in the substantia nigra of MPTP-treated PD mice (Mitra et al., 2016). The status of the non-canonical NF-κB activation signaling pathway in the 6-OHDA-lesioned striatum at a dyskinetic status remains unknown, which requires more experimental data to be testified.

Our studies showed that the inhibition of the BET protein function greatly blocked IKKα/β activation in the lesioned striatum, which was consistent to a previous report in a central nervous system inflammation model (Wang et al., 2018). Notably, IKKα/β activation is a cytoplasmic event (Perkins, 2007). The BET bromodomain is highly enriched in the specific regions of targeted genes. Theoretically, the inhibition of BET protein blocks the interaction between BET proteins and acetylates NF-κB in the nucleus (Zou et al., 2014; Gong et al., 2021). However, a report revealed that the inhibition of BET proteins blocked the activation of the p38 and JNK-MAPK signaling pathways, which might lead to the suppression on the IKKα/β activation (Wang et al., 2018; Sánchez-Ventura et al., 2019). NF-κB phosphorylation is crucial for its transactivation, which occurs at multiple p65 sites, including Ser 536 and Ser 276 (Chen et al., 2005; Brasier et al., 2011; Fang et al., 2014; Wang et al., 2014). Studies reported that activated IKKα/β directly phosphorylated p65 at Ser 536 and indirectly induced Ser 276 phosphorylation through the ATM-dependent pathway (Fang et al., 2014). Ser 536 and Ser 276 phosphorylation enhances the recruitment of p300, which thereby induces the acetylation of lysin 310 of p65 and the histones surrounding the targe genes (Chen et al., 2005; Brasier et al., 2011; Wang et al., 2014). We observed that intermittent L-dopa stimulation induced a hyperphosphorylation at Ser 536 and Ser 276 sites of p65 in the lesioned striatum of PD rats with serious dyskinesia movements. It might be attributed to the aberrant activation of IKKα/β, and inhibition of BET protein greatly suppressed IKKα/β activation, leading to a decrease in Ser 536 and Ser 276 phosphorylation.

The traditional implications on the mechanisms of LID were intermittent L-dopa stimulation triggered aberrant overresponse of the dopamine and non-dopamine receptors (NMDAR, etc.) in the striatal D1R-expressing medium-sized spiny neurons (MSNs), mediating its downstream signaling pathway to induce ERK1/2 phosphorylation (Picconi et al., 2018). Phosphorylated ERK1/2 further activated MSK1 and induced cAMP response element-binding protein (CREB) and H3 phosphorylation that stimulated gene expression, leading to the abnormal synaptic plasticity (Feyder et al., 2011; Beck et al., 2019). A recent study reported that BET proteins physically bound to the promotor and enhancer regions of the immediate early genes (IEGs) of fos b and arc (Figge and Standaert, 2017), which were enhanced by intermittent L-dopa stimulation. The inhibition of the BET protein function by JQ1 significantly blocked L-dopa-induced expression of IEGs and alleviated LID. Our findings indicated a novel role of BET protein in the mechanisms of LID by participating in the NF-κB-mediated neuroinflammation in the striatum. As ERK 1/2 phosphorylation was a crucial upstream event prior to the IEG transcription, we speculated that JQ1-blocked ERK 1/2 phosphorylation might be attributed to its suppression of the NF-κB-mediated gene expression of TNF-α and IL-1 β. With intermittent L-dopa stimulation, an increased cortico-striatal synaptic glutamate elicited an elevation of glia-derived TNF-α, which further enhanced the release of glutamate from the cortical neurons in a self-reinforcing manner (Dos Santos Pereira et al., 2021). The hyperactivity of cortico-striatal glutamatergic transmission positively contributed to the occurrence of LID by inducing neuron-expressed receptor-mediated ERK1/2 phosphorylation (Picconi et al., 2018). IL-1β was reported to activate the cortical neurons through the ionotropic glutamate receptor (N-methyl-D-aspartic acid receptor 2B, NR2B), mediating the downstream signaling pathway (Davis et al., 2006). Our recent study further confirmed that the expression level of TNF-α and IL-1 β positively correlated with the neuron membrane-expressed NR2B receptor-mediated ERK1/2 phosphorylation and the intensity of LID (Yan et al., 2021).

We confirmed that overactivation of the canonical NF-κB pathway mediated the striatal neuroinflammation of LID rat models, which was strongly suppressed by a BET inhibitor. Considering its anti-dyskinetic role and no interference with L-dopa treatment benefits, BET protein might be a potent intervention target for LID, and certainly, more experiments are required for future clarification of the role of BET protein in the mechanisms of LID.

## Data Availability Statement

The raw data supporting the conclusions of this article will be made available by the authors, without undue reservation.

## Ethics Statement

The animal study was reviewed and approved by the Institutional Review Board of Xinhua Hospital Affiliated to JiaoTong University, School of Medicine, Shanghai.

## Author Contributions

ZL and JG designed the study. SY created the sham and 6-OHDA PD rat models. YW and LH carried out the medication intervention and animal behavior assessment and wrote the manuscript. LR and LS performed RNA isolation, real-time PCR, ELISA test, protein extraction, and Western blot. NW conducted the data analysis. All authors contributed to the article and approved the submitted version.

## Conflict of Interest

The authors declare that the research was conducted in the absence of any commercial or financial relationships that could be construed as a potential conflict of interest.

## Publisher’s Note

All claims expressed in this article are solely those of the authors and do not necessarily represent those of their affiliated organizations, or those of the publisher, the editors and the reviewers. Any product that may be evaluated in this article, or claim that may be made by its manufacturer, is not guaranteed or endorsed by the publisher.
